# Predictive demographic and clinical features for the development of dysthyroid optic neuropathy in a multi-ethnic TED population: a retrospective cohort study

**DOI:** 10.1186/s13044-025-00249-4

**Published:** 2025-07-22

**Authors:** Malik Moledina, Vickie Lee, Ahmed Alnahrawy, Ourania Fydanaki, Nicole George, Nour Hubby, Daisy Metcalf, Natalie Man, Gabriella Guevara, Claire Feeney, Rashmi Akshikar, Rajni Jain, Ahmad Aziz, Vassiliki Bravis, Karim Meeran

**Affiliations:** 1 Oculoplastics and Adnexal Service, Western Eye Hospital, Imperial College NHS Foundation Trust, 53-173 Marylebone Rd, London, NW1 5QH UK; 2 Imperial College Ophthalmology Research Group (ICORG), London, UK; 3 Department of Metabolic Medicine, St Mary’s Hospital, Imperial College Healthcare NHS Foundation Trust, London, UK; 4 Faculty of Medicine, Department of Metabolism, Digestion and Reproduction, Imperial College London, London, UK; 5 National Institute of Health Research Imperial Clinical Research Facility, London, UK

**Keywords:** Dysthyroid optic neuropathy, Thyroid eye disease, Graves orbitopathy, Risk factors

## Abstract

**Background:**

Dysthyroid Optic Neuropathy (DON) is a sight-threatening complication of Thyroid Eye Disease (TED). This study aims to identify the risk and predictive factors for DON in a multi-ethnic TED cohort.

**Methods:**

Retrospective, cohort study of consecutive TED patients attending a multidisciplinary service over an 11-year period. Consecutive patients aged over 18 years old with a minimum of 6 months follow-up post-diagnosis of TED were included. We compared those patients with DON and those without (no-DON) to determine which factors were more prevalent in patients with DON.

**Results:**

There were 26 and 516 consecutive patients with DON and no-DON. The DON prevalence in the cohort was 5.0%. The DON group had a Mean Age at TED Diagnosis (MATD) of 57.8 vs. 46.1 years in the no-DON group. The mean presenting CAS, TRAb and Gorman Diplopia Score (GDS) were significantly higher 3.73 ± 1.80, 2.76 ± 1.05 and 11.31 ± 11.90 vs. 0.54 ± 0.80, 0.48 ± 0.90 and 6.95 ± 9.22 in the DON compared to the no-DON group respectively (*p* = 0.00, *p* = 0.00 and *p* = 0.04). On multivariable regression, we found the following risk factors for developing DON (Odds Ratios): MATD ≥ 53 years (5.2 *p* = 0.00), presenting CAS ≥ 4 (*P* = 0.00), presenting GDS ≥ 3 (7.5 *p* = 0.00), diabetes (5.7 *p* = 0.00), and baseline TRAb ≥ 5.0 IU/L (2.9 *p* = 0.04).

**Conclusion:**

Patients with diabetes, increased MATD, and high presenting CAS, GDS, and TRAb are at increased risk of developing DON in our cohort. Clinicians should be especially vigilant of the risk of sight-threatening complications in TED patients with more than one of the above risk factors.

## Background

Thyroid Eye Disease (TED) is an autoimmune condition with orbital inflammation associated with Graves’ Hyperthyroidism (GH). Previous studies have found that 20–50% of GH patients develop TED [1]. One of the most consequential complications of TED is Dysthyroid Optic Neuropathy (DON), which can result in severe and irreversible visual impairment in 3–5% of patients [2–4]. The pathophysiology of DON is secondary to an autoimmune-mediated inflammation causing volume expansion of the orbital tissues within the confined orbital cavity. This can lead to secondary vascular and mechanical compromise of the optic nerve due to extraocular muscle (EOM) enlargement at the orbital apex [3]. Early diagnosis and management of DON are critical to achieving a good visual outcome [5]. A well-recognized challenge is the lack of a gold-standard diagnostic algorithm. Controversy surrounds which diagnostic parameters should be given the most consideration, especially in the identification of atypical or early DON [5].

There are limited peer-reviewed large cohort studies evaluating the risk factors for developing DON [6–8] Our study aims to provide an in-depth comparative analysis of the predictive factors and characteristics of DON patients uniquely in a multi-ethnic TED population from a large metropolitan tertiary centre. We aim to assess novel risk factors such as presenting TRAb levels and diplopia, as defined by Gorman Diplopia Scores, that have not yet been evaluated in the context of DON. We also aim to understand how the application of GoQoL relates to DON patients.

## Methods

This study is an anonymised retrospective, comparative study of patients within a multidisciplinary TED (MDTED) service covering two large metropolitan NHS hospital clusters. We included consecutive TED patients diagnosed within an 11-year period between 2011 and 2022. Patients were jointly managed by TED-specialist oculoplastic consultants (VL, AA, RJ) and consultant endocrinologists (KM, VB and CF) alongside technicians for imaging, visual field and ocular motility assessment. An immunosuppression specialist (RA) advised on non-steroidal immunosuppression treatment. The study was approved by the audit department and adhered to the Declaration of Helsinki and all laws in the United Kingdom.

Extraction of data was via a TED database populated contemporaneously following each clinic visit. The data that was prospectively collected at each clinic visit included: the thyroid diagnosis (GH, hypothyroidism, Hashimotos or euthyroid) and thyroid status (euthyroid, hyper or hypothyroid). The treatment the patient had undergone (medical management, radio-iodine (RAI), thyroidectomy or no treatment), TED severity (EUGOGO criteria: mild, moderate-severe or sight-threatening), Gorman diplopia score [9], clinical activity score: CAS whereby a 7-item CAS was utilised for the baseline and a 10-item CAS for subsequent follow-up visits [10], the quality of life Assessment: GoQoL [11] were recorded. Previous and current treatments, including systemic immunosuppression, orbital radiotherapy and orbital decompression surgery, were also recorded. Active hyperthyroidism was defined as an elevated T3 or T4 level above the normal range. Further retrospective data collection included patient demographics (age at TED diagnosis, ethnicity, gender, smoking status), clinical parameters (clinical history, diabetes mellitus (DM) status, visual acuity, RAPD status, colour vision, exophthalmometry) and results of investigations (MRI radiological parameters, visual field assessment and TRAb). The TRAb utilised is a human monoclonal autoantibody to the TSHR identifying blocking and stimulatory antibodies, labelled with biotin (M22-Biotin) where < 0.4 IU/Litre is considered negative).

**Fig. 1:**
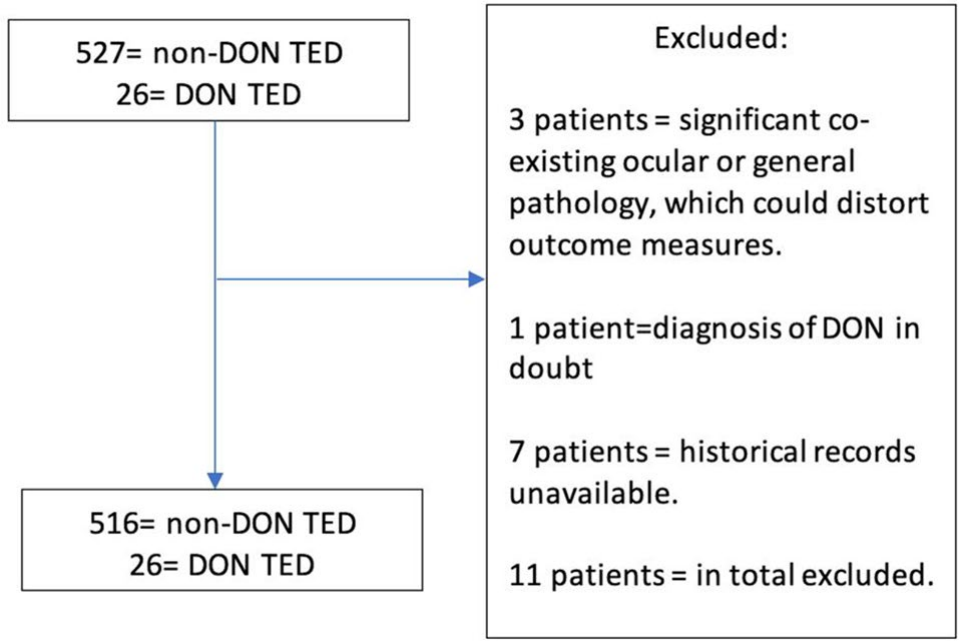
Strobe diagram depicting excluded participants

Inclusion criteria for the study: A patient age  ≥ 18 years with a clinical diagnosis of TED AND a minimum of 6 months follow-up following diagnosis. Exclusion criteria were: endocrinology input not based at either site and/or diagnosis of TED/DON in doubt, and/or co-existing ocular or general pathology, which could confound outcome measures and natural history, and/or data unavailable (Fig. 1).

We utilised a modified criteria as per Khong et al and Dayan et al. to define DON [7, 12]. This included **two** of the following criteria: (1) decline of visual acuity of ≥ 5 letters, (2) reduction in colour vision, (3) a positive Relative Afferent Pupillary Defect (RAPD), (4). optic disc swelling/abnormalities (without an alternative causes), (5). MRI changes consistent with DON [13], and (6). Visual field (VF) changes correlated with DON (utilizing Mean Deviation, common identifiable VF patterns commonly found and ascribable to DON [14, 15] without an alternative cause and correlation to orbital imaging) AND **without** other attributable causes, e.g. cataract, ocular surface changes, retinal disease. Confounding variables were jointly reviewed, and their contributions and impact on visual assessment were assessed. Where any doubt existed as to the contribution such confounders had on the decline in vision, these cases were excluded [12]. Baseline vision was determined from previously available written or electronic records or recent assessments from an allied health professional, e.g., an optometrist visit. On diagnosing DON, clinical judgement was applied with the utililisation of clinical tools e.g Callahan et al where appropriate [16].

The standard of care for our TED patients is outlined in the EUGOGO (consensus) guidelines [17]. For DON patients, 1 g intravenous methylprednisolone (IVMP) infusions administered over three days were performed with subsequent review. Treatment responders were moved onto the EUGOGO 12-week IVMP protocol in conjunction with an approved second-line agent. Usual second-line treatments included a 12–18 month course of mycophenolate mofetil (MMF) and/ or orbital radiotherapy. Non-responders or flares (defined as a deterioration of any of the DON diagnostic parameters) on immunosuppression underwent an endonasal medial wall orbital decompression and then were treated with the same regimen as above. Patients remain in the MDTED service until rehabilitative surgery, when they are transferred to the general oculoplastic surgical services, so the last follow-up visits recorded for this study are prior to any elective surgical intervention.

The study approved by the institutional audit department was conducted by the tenets of the Declaration of Helsinki and all laws in the United Kingdom. A distinct analysis focused on the MRI Findings in DON, utilising sections of this dataset has been published elsewhere [18].

### Statistical analysis

Patients were divided into two cohorts: those with DON and those without (no-DON). With continuous variables, normality was assessed using a histogram plot, Shapiro-Wilk and Kolmogorov-Smirnov Tests. Where normality existed (e.g. age), an independent t-test was performed; otherwise, non-parametric equivalents were utilised, such as a Mann-Whitney U Test (e.g. TRAb and GoQoL). Categorical data (all other variables) were compared using Pearson’s Chi-Square/Fisher’s exact test. When a variable was < 5, Fisher’s Exact Test was performed. Univariable and multivariable binary logistic regression analyses were performed on all factors that were statistically significant and likely predictive of DON. Factors initially underwent univariable analysis (UVA), with those achieving significance proceeding to multivariable regression (MVA).We also performed testing for multi-collinearity for predictor variables, utilising Variance Inflation Factor (VIF), which were all within tolerable limits (< 3), and all tolerance values were above 0.2. There was no evidence of any strong correlation or association between the predictor variables when tested. We were, therefore, not concerned that any multi-collinearity was present. On univariable and multivariable binary logistic regression analysis, the omnibus tests of model coefficients were found to be significant compared to the null model, whilst the Hosmer and Lemeshow test was insignificant, suggesting the model showed a good fit for the data. Differences were considered statistically significant, where *p* < 0.05. Analyses were performed with SPSS V.24.0 (IBM SPSS Statistics for Macintosh).

## Results

There were 26 and 516 consecutive patients with DON and no-DON. The mean follow-up at review for the DON group was 30.5 months compared to the Non-DON group, which was 25.2 months. The DON prevalence was 5.0%, with 84.6% (22/26) with bilateral DON in the cohort. In the DON cohort, 19.2% (5/26) patients were of Caucasian ethnicity and 19.2% (5/26) patients were male, compared to 34.7% (152/438) and 22.2% (105/474) in the non-DON group, respectively (*p* = 0.105 and *p* = 0.474). In the DON cohort, 26.9% (7/26) were active smokers, and 36% (9/25) had uncontrolled hyperthyroidism compared to 25.7% (115/447) and 29.5% (150/509) in the non-DON contingent, respectively (*p* = 0.565 and *p* = 0.486). The percentage of patients diagnosed with Graves’ Disease, prior radioiodine exposure and diabetes were 92.3% (24/26), 21.1% (4/19) and 26.9% (7/26) respectively in the DON group compared to 90.9% (469/516), 15.0% (66/440) and 8.3% (30/362) respectively in the non-DON group (*p* = 1.000, *p* = 0.528 and *p* = 0.007) (Table 1).

**Table 1:**
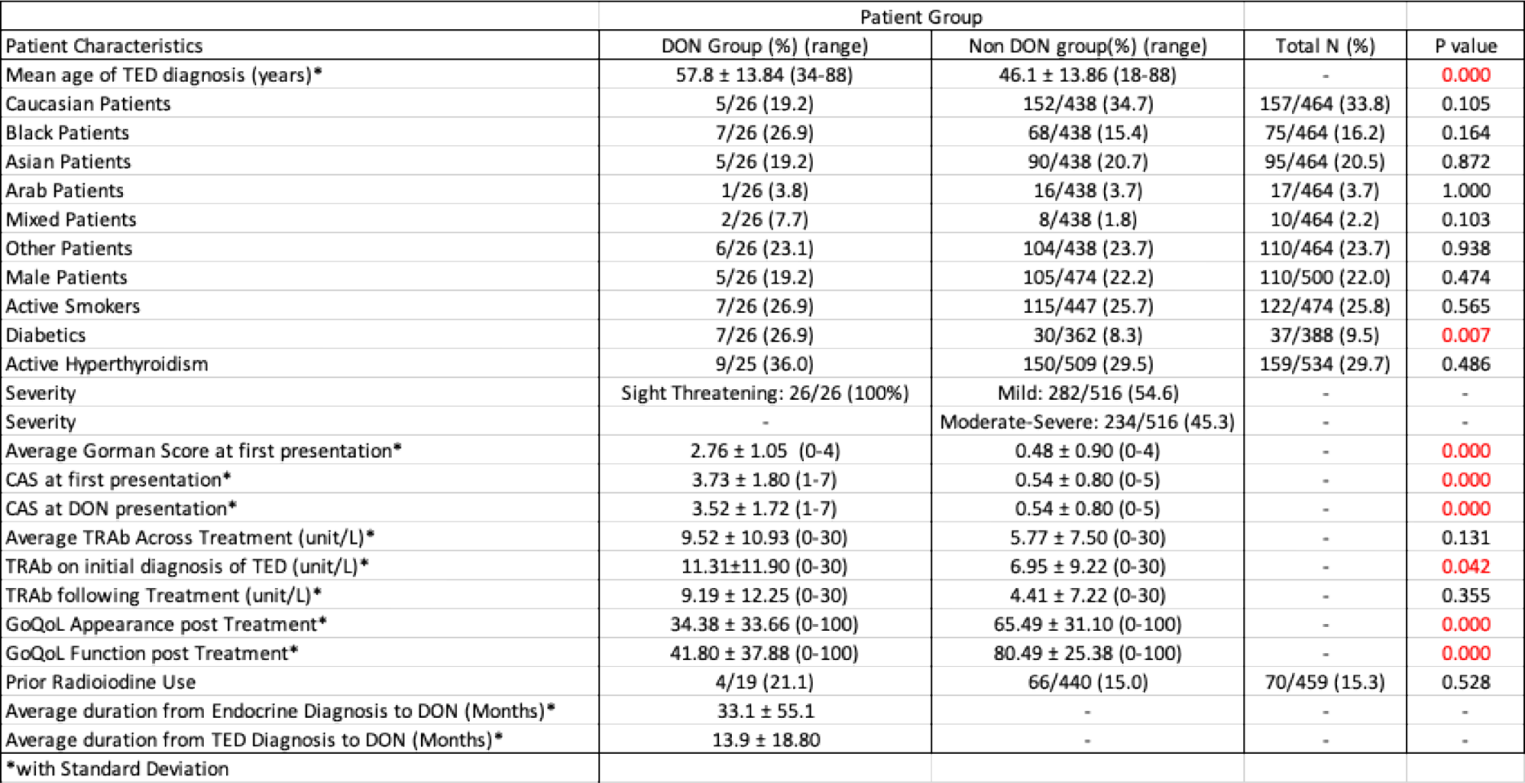
Demographic, clinical and baseline characteristics of DON and non-DON TED patients

In the DON group, 92.3% (24/26) of patients were started on steroid treatment, with only two patients where this was contraindicated. Mycophenolate mofetil was utilised in 65.4% (17/26), 30.8% (8/26) were treated with orbital radiotherapy and 7.7% (2/26) were treated with Ciclosporin; Urgent orbital decompression surgery was undertaken in 53.8% (14/26) of patients.

The mean presenting CAS at TED diagnosis was 3.7 (± 1.8) in the DON group compared to 0.5 (± 0.8) in the no-DON group (*p* < 0.001). The mean CAS at DON diagnosis was 3.5 (± 1.7) in the DON group. The mean presenting diplopia score (Gorman) in the DON group at TED diagnosis was 2.8 ± 1.1 (SD) compared to 0.5 ± 0.9 in the no-DON group (*p* < 0.001). The mean GoQoL appearance score at the last follow-up visit in the DON group was 34.4 ± 33.7 compared to 64.5 ± 31.1 in the no-DON group (*p* < 0.001). The mean GoQoL visual function score at the last follow-up visit in the DON group was 41.8 ± 37.9 compared to 80.5 ± 25.4 in the no-DON group (*p* < 0.001).

The mean TRAb at TED diagnosis for the DON cohort was 11.3 (± 11.9) compared to 7.0 (± 9.2) for the non-DON cohort (*p* < 0.05). The mean TRAb following treatment in the DON cohort was 9.2 (± 12.3) compared to 4.4 (± 7.2) in the no-DON group (*p* > 0.05).

**Fig. 2:**
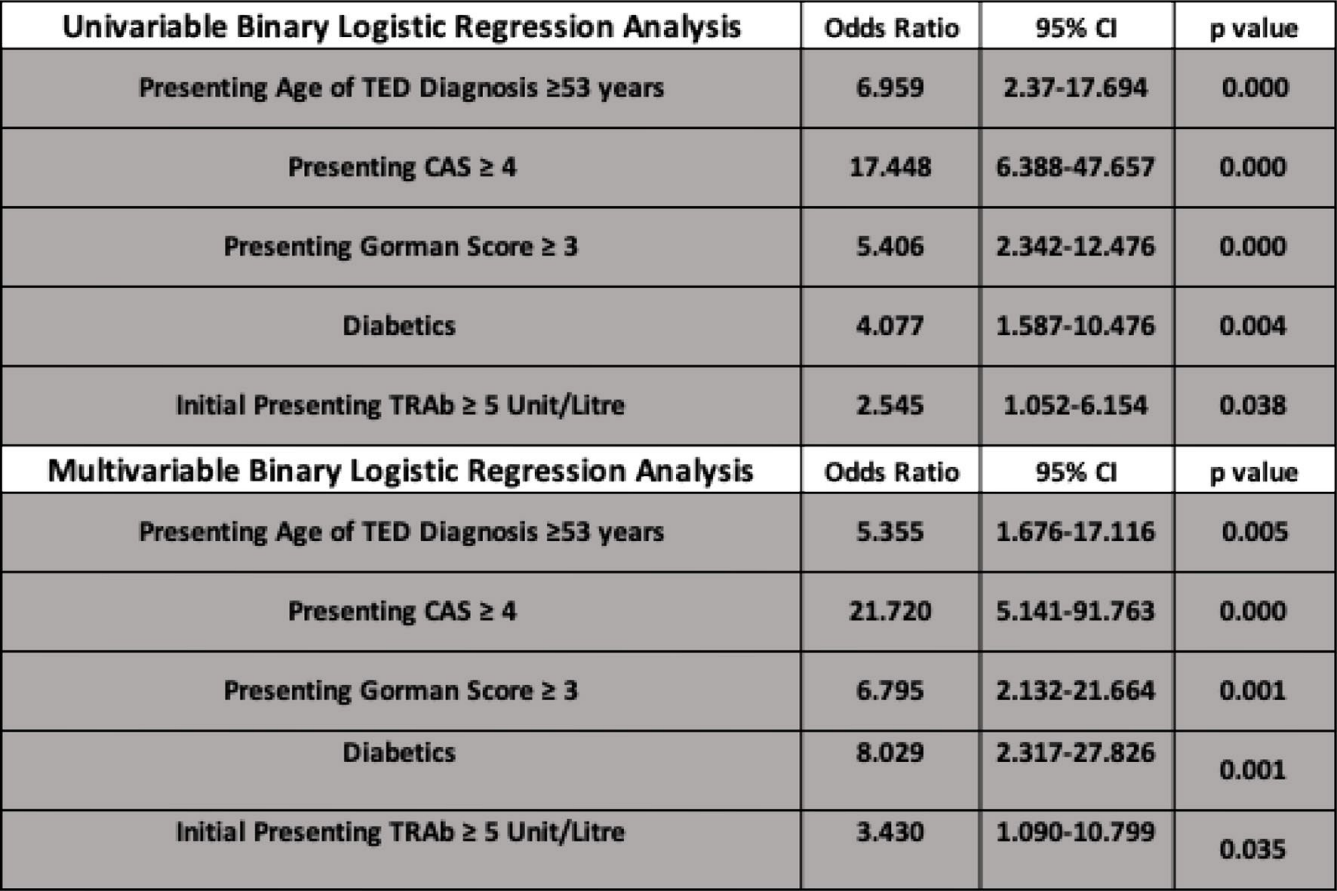
Univariable and multivariable binary regression analysis of DON risk factors

**Fig. 3:**
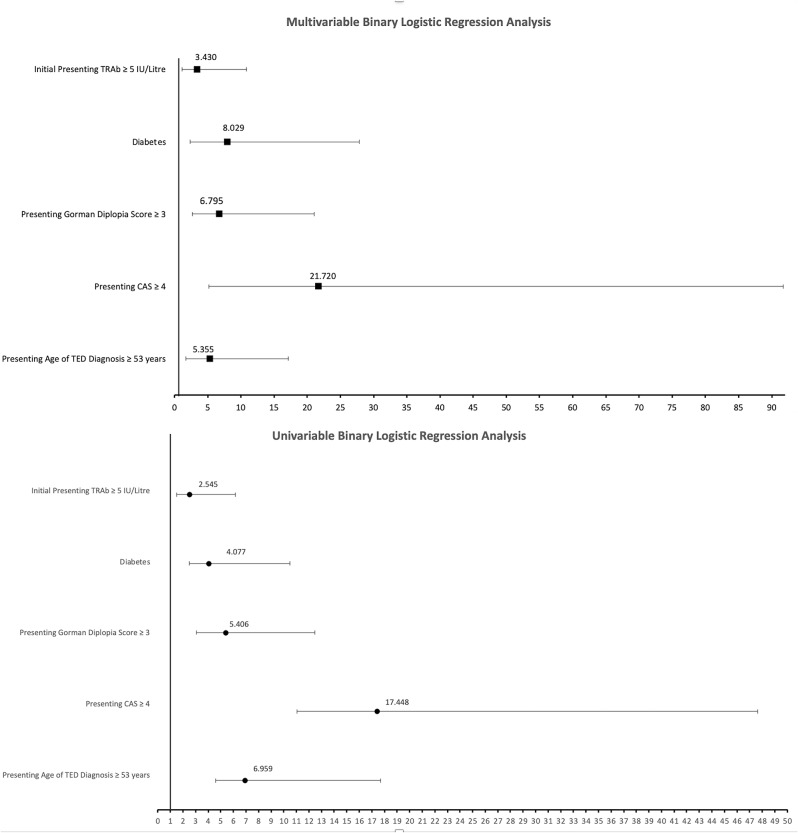
Univariable and multivariable binary regression analysis of DON risk factors represented as odds ratio plot (bottom)

We identified the following risk factors for the development of DON on univariate and multivariate analysis: MATD ≥ 53 years, presenting CAS ≥ 4, presenting diplopia score (Gorman) ≥ 3, DM, and baseline TRAb ≥ 5 IU/Litre (Figs. 2 and 3).

## Discussion

Our study provides a comparative analysis of the predictive and risk factors for developing DON in a multi-ethnic metropolitan TED cohort.

### Age at diagnosis of TED AATD

We found a ten-year difference in MATD between DON and no-DON cohorts, with MATD ≥ 53 years having a 5.4 times increased odds of developing DON on multivariate regression. Our finding is relative to other studies with different ethnic make-ups. One study with a predominantly east asian cohort found a threefold increase in DON risk at an older age (≥ 55 years) [8]. Another multicenter study, with a predominantly Caucasian cohort, found a 1.58 increased risk of DON with each decade rise in age of TED diagnosis [7]. Some authors have postulated this may be due to a greater prevalence of co-existing vascular disease [19]. Nevertheless, one study found an increasing prevalence of DON in older patients, but this failed to reach statistical significance [20]. Another large study from Germany failed to demonstrate age as a risk factor. This study by Oeverhaus et al. highlighted that gender and smoking status had been omitted from some studies, which showed older age to be associated with a greater risk of DON and consequentially, this may have influenced the results [21]. Despite this, in our study, we failed to demonstrate gender or smoking status as influencing the risk of developing DON.

### Diabetes mellitus (DM)

There are conflicting reports in the literature regarding the role of diabetes in TED [6, 22–25]. In our study, 25% of DON patients had DM, similar to other studies that found DM prevalence at 25% and 19.23% [6, 26]. We also found our TED DM patients had almost 8 times the risk of developing DON (*p* = 0.00). Other studies have observed patients with diabetes are at higher risk for recurrent DON and less responsive to conventional treatment [8, 22, 23]. Possible mechanisms may involve increased IGF-1 production in DM stimulating the co-located TSH-IGFR-1 receptor, leading to increased TED orbital inflammation [27]. DM-related micro-vasculopathy may also increase the risk of optic nerve hypoxia [8, 28]. We recommend that all patients with DON have an HbA1c checked, and appropriate escalation in diabetes treatment for those found to have diabetes or pre-diabetes arranged with the introduction of glucocorticoid treatment. Clinicians may also consider the set-up of their services and opt to develop a Multi-Disciplinary Team (MDT) clinic set-up involving a consultant endocrinologist and an immunosuppression specialist. This allows confident treatment of patients with medical co-morbidities, medical optimisation and excellent monitoring prior to and during immunosuppression and has been shown to improve outcomes for TED patients [17, 29].

### Clinical activity score (CAS)

We found that a high CAS at TED diagnosis in our DON cohort (3.73 ± 1.80) increased the risk of developing DON by almost 22 times (*p* = 0.00). This trend was also observed in several other studies [7, 25, 30]. However, several patients who were diagnosed with DON, were found to have a clinically inactive CAS score at the time of diagnosis of DON. Furthermore, two patients had a CAS score of 1, and both of these patients were non-Caucasians with darker skin tones, one of which had a crowded apex on MRI imaging. This has been reported elsewhere. In the DON EUGOGO study, a quarter of patients with sight-threatening disease had CAS < 3 [30]. A significant limitation of the CAS score is that it was developed in a primarily Caucasian cohort, and several of the anterior orbital signs may be less reliable in non-Caucasian populations. Furthermore, the CAS Score is heavily skewed towards anterior orbital signs despite some phenotypes of TED predominating in a retrobulbar location [10]. A study by Uddin et al. characterised the heterogeneity of TED phenotypes [31]. One phenotype coined, the “white eye apex” typically presents as DON with few anterior orbital signs but on MRI imaging, is found to have a crowded apex with relatively more space in the anterior two thirds of the orbit. Clinicians should, therefore, remain vigilant and appreciated that patients may have DON despite having inactive CAS scores [31].

### Thyroid-receptor antibodies (TRAb)

TRAb is the only biomarker for TED, though several assays exist, and is also widely used in predicting the relapse of GH. Other published studies have found TRAb titres correlate with CAS so it may be a possible predictor for future TED disease severity [32–35]. A study by Stohr et al. evaluated several antibody assays to assess their predictive power in determining the course of GO at different time points. Patients were divided into a mild course as classified by a NOSPECS, score < 5 and a severe course with a NOSPECS score > 5. The study calculated a variety of threshold values that were related to a specificity of detection of 90%. The authors found all assays had a similar degree of sensitivity, but interestingly, sensitivity increased after five months of onset of GO. They concluded that TRAb levels provide additional information in the risk stratification of patients into a mild or severe course [36].

Nevertheless, to our knowledge, presenting TRAb levels have not yet been investigated directly in relation to DON. We found that our DON group had a higher baseline TRAb (*p* = 0.04) at presentation compared to the no DON group. On multivariate analysis, we found that TED patients presenting with a baseline TRAb ≥ 5 IU/Litre had an OR 3.4 x of developing DON (*p* = 0.04). This may have clinical relevance in risk stratifying patients on presentation at risk of developing DON at a later stage. Future studies should investigate how this threshold may change over the course of the disease through serial measurements and whether this impacts the risk of developing DON.

### Gorman diplopia scoring GDS

Studies have shown patients with DON have a greater propensity of having diplopia. Only one other study has evaluated clinical strabismus as a predictive feature for developing DON. This study evaluated strabismus in terms of the Hirschberg test [7, 30]. Our study has been the first to evaluate diplopia and its relationship to the development of DON utilising the Gorman score, a widely used, efficient and effective grading system for diplopia in the clinical setting. We found on multivariate analysis patients with constant diplopia (GDS 3) at TED diagnosis had an odds ratio of 7 of developing DON (*p* = 0.00). This can be correlated with the structural enlargement of the extraocular muscles, particularly the medial rectus (MR) and the superior muscle complex. An imaging study investigating EOM enlargement in DON found an increase in the volume of the MR to be the strongest predictor in the development of DON in TED patients [37]. Other studies have corroborated these findings, with some authors postulating the MR’s close anatomic position to the optic nerve and canal being responsible for this finding, with others suggesting this may be due to the MR having a greater predisposition to inflammatory changes [37–39] One study has implicated the superior oblique enlargement as the strongest predictor for DON. Although the SO is unlikely to cause direct compression of the optic nerve, the authors suggest SO enlargement may be a proxy for more severe disease and, as a consequence a greater likelihood of developing DON [38]. Enlargement of the EOM’s particular the medial rectus and superior muscle complex can lead to diplopia and increase DON risk due to crowding at the orbital apex [15, 39]. 

### Quality of life scores (Go-QoL)

**Fig. 4:**
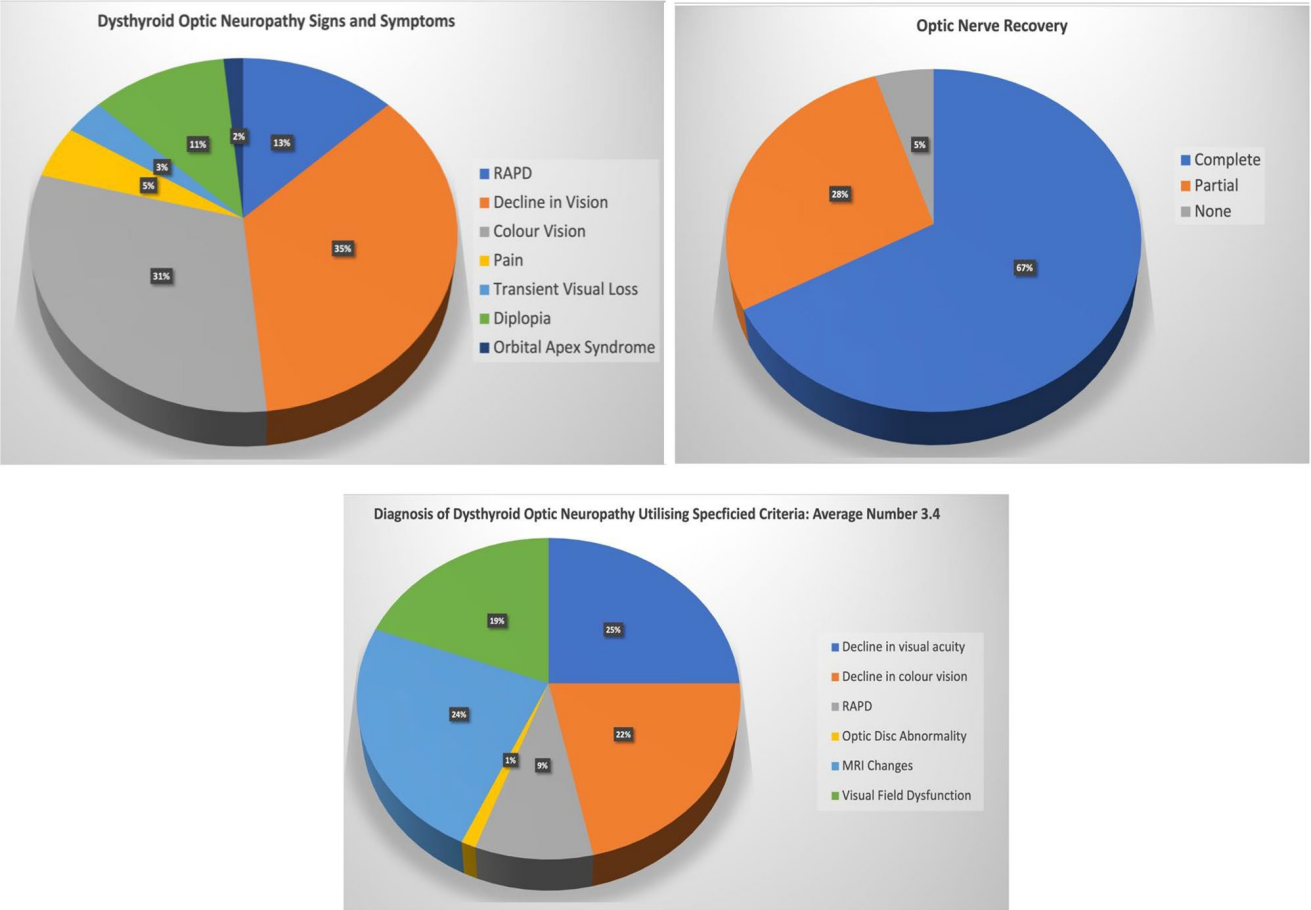
Top: Pie chart of presenting signs and symptoms during the diagnosis of DON, Middle: Proportion of patients recovered following treatment of DON. Bottom: Pie Chart identifying multiple criteria used to diagnose DON with an average number of 3.4 per patient

The Go-QoL is the only validated questionnaire for all levels of TED severity, but the literature is sparse when evaluating GoQoL in DON [40]. Our study found a significantly lower GoQoL (> 30 points) at the last follow-up for both the visual function and appearance scores in the DON relative to the no-DON cohort. This is in the context of previously validated studies recommending a difference of 10 points should be considered clinically significant [11]. We identified 67% of post-DON patients in this study to have improved visual acuity and Ishihara colour vision back to ‘normal’ following treatment (Fig. 4). This discrepancy between our objective assessments and patient-reported subjective GoQoL measures are a novel finding in DON. The poor GoQoL visual function scores is likely to reflect the recognised discordance between objective clinical assessment and a patient’s lived experience of TED and may include subtle changes in colour vision particularly impacting tritan colours that Ishihara colour vision testing is not sensitive in detecting [41]. The persistence of diplopia in many patients may also be reflected in the low visual function scores. Moreover, it is well established that conventional immunosuppression does not improve proptosis [42] so continues to be reflected in the low GoQoL appearance scores before rehabilitation surgery.

### Other risk factors

Our study did not identify gender or ethnicity as DON risk factors. Smoking is an established risk factor for developing TED [43], and DON in some studies [8]. Our finding that smoking status did not appear to elevate DON risk correlates with a large Australian multicenter study, looking primarily at Caucasians, evaluating 1042 patients that found smoking status to be associated with increased odds of TED but not with DON [7]. The study also found no correlation between smoking status and severity in ocular motility restriction [7]. However, another study evaluating Korean patients found smoking to be the most significant risk factor in the development of DON [44]. The variation and heterogeneity of both the compounds found in cigarette smoke, over 4000, and their concentration often makes comparisons challenging and may explain the contrasting results [45]. Furthermore, in our study, we evaluated patients based on self-identification of being a smoker, non-smoker or ex-smoker. This was not confirmed via a urine cotinine test, nor did we quantify in pack years the degree to which patients were smokers or ex-smoker which may have influenced our results.

The pathogenesis behind smoking and its impact on TED is complex and not fully understood. It is thought that cigarette smoking impacts both the adaptive and innate immune systems as well as direct action on fibroblast activity, resulting in the propagation of TED [46]. One study evaluated the impact of cigarette smoke on ICAM-1 expression and found a lack of effect but a substantial impact on IL-1 expression, suggesting the pathogenesis may predominantly act via the adaptive immune system. It is becoming apparent that ethnic variations may influence the course of TED, and differences in the adaptive immune response may be responsible for this. Studies have corroborated this by showing the utility of different antibody markers’ in TED for different populations [47, 48]. It is possible, that the degree of adipogenesis, fibroblast activation and inflammation resulting in apical crowding and, subsequently, DON, may be influenced by differences in adaptive immunity responses determined by genetic factors and susceptibility.

Previous studies have shown that optimising thyroid status may reduce the chances of developing TED and also reduce severity [47], whilst others have shown no association [44, 49]. Our study did not find that a hyperthyroid status at TED diagnosis increased the risk of DON. The use of RAI in the treatment of GH is recognised as a risk factor for TED de-novo onset or exacerbation [17], and in some studies increases the risk of DON [30, 50, 51]. Our study did not show previous treatment with RAI increased the risk of DON and corroborates the findings from the Australian multi-centre study where TED risk following RAI was reduced from an odds ratio of 2.37 to 1.37 when other variables were controlled for in the multivariate analysis [7]. The literature has also shown the benefits of steroid pre-treatment prophylaxis prior to RAI administration. This could be the difference between a higher risk of DON and no elevated risk and may also explain the lack of a statistical difference in our study [29]. Nevertheless, it should be noted that our study, due to the relatively small number of DON patients despite the large overall cohort, has not been sufficiently powered to show such a difference.

### Limitations

Our study is retrospective, non-blinded, with all inherent limitations that include missing data including inadequate visual field data. All available data was included in the analysis. As far as we are aware, our study is the first to analyse the risk of presenting TRAb titres in a large multi-ethnic patient cohort.

## Conclusion

We recommend increased vigilance for DON in TED patients with high presenting CAS and Gorman scores, higher TRAb titres, diabetes, and older age at TED diagnosis. Despite improved objective markers following DON treatment, many patients continue to have persistently low GoQoL scores.

## Data Availability

The datasets used and/or analysed during the current study are available from the corresponding author upon reasonable request.
